# Supplementary material for: Chimeric Peptide Engineered Nanomedicine for Synergistic Suppression of Tumor Growth and Therapy-Induced Hyperlipidemia by mTOR and PCSK9 Inhibition
Source: Pharmaceutics. 2023 Sep 23;15(10):2377. doi: 10.3390/pharmaceutics15102377 (PMC10610039; doi:10.3390/pharmaceutics15102377)
Supplement: Supplementary file 1 [file pharmaceutics-15-02377-s001.zip › pharmaceutics-2580012-supplementary.pdf]

pharmaceutics

Supplemental Materials

Chimeric Peptide Engineered Nanomedicine for Synergistic Suppression of Tumor Growth and Therapy-Induced Hyper-lipidemia by mTOR and PCSK9 Inhibition

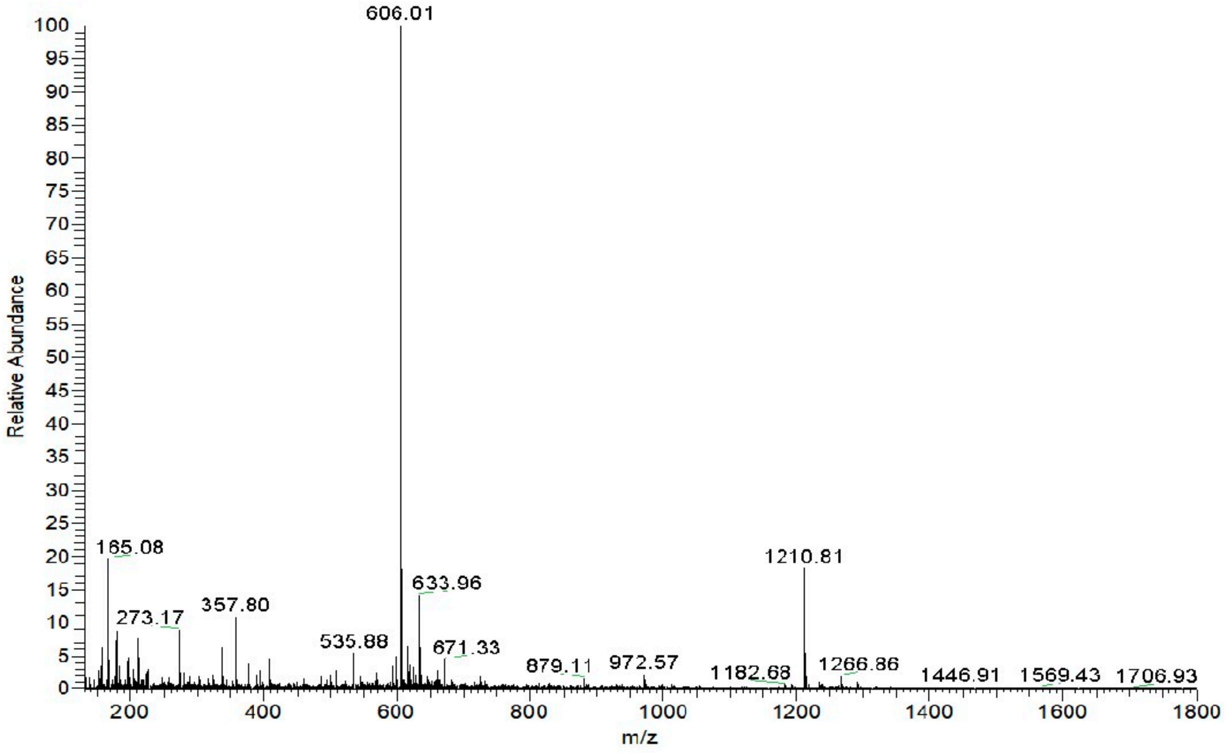

Figure S1. The mass spectrum of P nanoparticle.

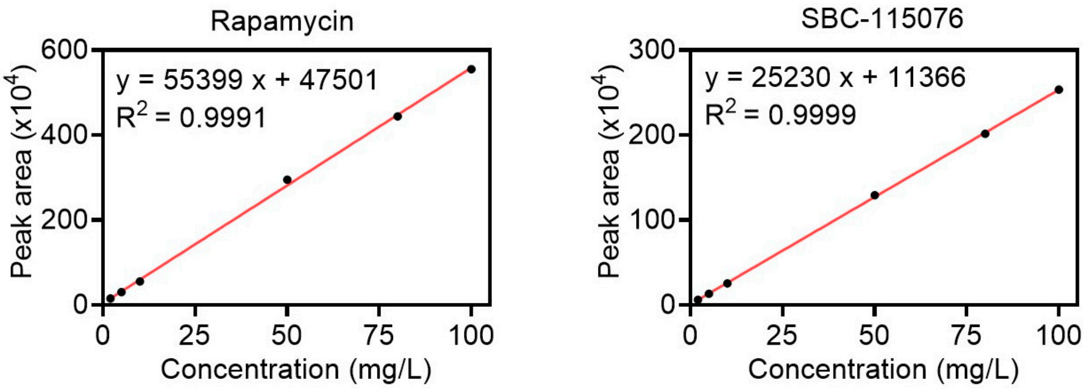

Figure S2. Linear equation between peak areas and concentrations of Rapamycin and SBC-115076 components by HPLC.

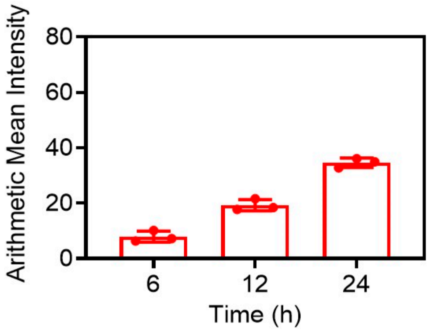

Figure S3. The arithmetic mean Intensity analysis of CLSM images of 4T1 cells after treatment with Cy5.5-PRS in the equivalent amount of Rapamycin (10 mg/L) for 6, 12 and 24 h.

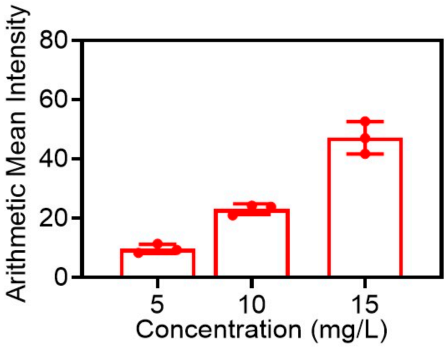

Figure S4. The arithmetic mean intensity analysis of CLSM images of 4T1 cells after treatment with Cy5.5-PRS for 24 h in the equivalent amount of Rapamycin (5, 10 and 15 mg/L).

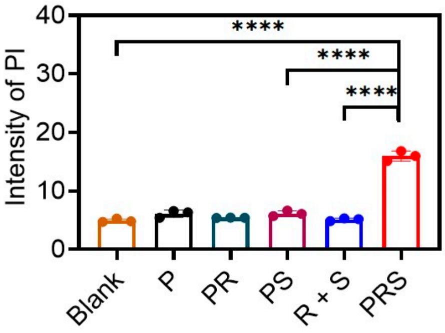

Figure S5. The mean intensity analysis of CLSM images of 4T1 cells by liver/dead cell staining assay. R + S represented the combined administration of Rapamycin and SBC-115076. ****P <0.0005 was tested via a Student’s t test.

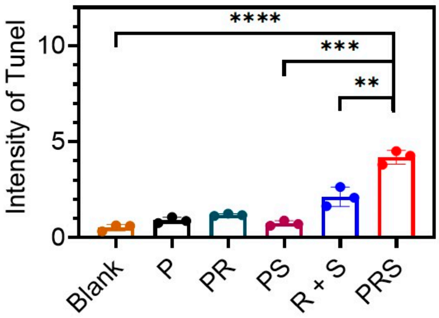

Figure S6. The mean intensity analysis of CLSM images of 4T1 cells by Tunel immunofluorescence. R + S represented the combined administration of Rapamycin and SBC-115076. ****P <0.0005 was tested via a Student's t test.

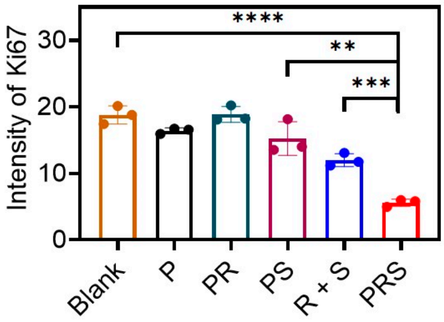

Figure S7. The mean Intensity analysis of CLSM imagesof 4T1 cells by the Ki67 immunofluorescence. R + S represented the combined administration of Rapamycin and SBC-115076. ****P <0.0005 was tested via a Student's t test.
